# Robot-assisted resection of choledochal cysts in children weighing less than 6 kg

**DOI:** 10.1093/bjs/znac361

**Published:** 2022-10-26

**Authors:** Yi Jin, Qingjiang Chen, Yuebin Zhang, Duote Cai, Wenjuan Luo, Shuhao Zhang, Di Hu, Zhigang Gao

**Affiliations:** Department of General Surgery, Children's Hospital, Zhejiang University School of Medicine, National Clinical Research Centre for Child Health, Hangzhou, China; Department of General Surgery, Children's Hospital, Zhejiang University School of Medicine, National Clinical Research Centre for Child Health, Hangzhou, China; Department of General Surgery, Children's Hospital, Zhejiang University School of Medicine, National Clinical Research Centre for Child Health, Hangzhou, China; Department of General Surgery, Children's Hospital, Zhejiang University School of Medicine, National Clinical Research Centre for Child Health, Hangzhou, China; Department of General Surgery, Children's Hospital, Zhejiang University School of Medicine, National Clinical Research Centre for Child Health, Hangzhou, China; Department of General Surgery, Children's Hospital, Zhejiang University School of Medicine, National Clinical Research Centre for Child Health, Hangzhou, China; Department of General Surgery, Children's Hospital, Zhejiang University School of Medicine, National Clinical Research Centre for Child Health, Hangzhou, China; Department of General Surgery, Children's Hospital, Zhejiang University School of Medicine, National Clinical Research Centre for Child Health, Hangzhou, China

*Dear Editor*

Choledochal cyst is a congenital dilatation of the biliary system. The current treatment for this is cyst excision with Roux-en-Y hepaticoenterostomy^1^. The detection of prenatal choledochal cysts is increasing with the improvements in antenatal detection, but the timing of surgery after birth remains controversial. If the cyst size increases significantly or repeated inflammation occurs during observation, early surgery is considered necessary^2^. With the development of minimally invasive technology, the invention and application of a robotic surgical system has created a new era of surgery^3^. Ihn *et al*.^4^ reported that a bodyweight of 6 kg was one of the exclusion criterion for robot-assisted surgery, whereas Xie *et al*.^5^ suggested that robotic surgery was not recommended in children aged less than 6 months. This retrospective study aimed to verify the feasibility of this procedure for patients weighing less than 6 kg.

A total of 10 patients weighing less than 6 kg with choledochal cysts who had undergone robot-assisted surgical resection between April 2020 to April 2022 were studied retrospectively (*Table 1*).

**Table 1:** Patient clinical data

Patient	Sex Age (years)	Weight (kg)	Prenatal examination finding	Cyst type	Cyst diameter (cm)	Duration of surgery (min)	Diameter of hepatojejunal anastomosis (cm)	Intraoperative blood loss (ml)	Postoperative feeding time (days)	Postoperative hospital stay (days)
1	F 1m 12d	3.5	No	Ia	7	248	1.2	5	5	9
2	F 1m 13d	5.2	Yes	Ia	3.4	210	1	5	5	8
3	F 2m 17d	5.3	Yes	Ia	1.7	220	1.3	2	3	15
4	F 1m 13d	5.5	Yes	Ia	2.5	175	1	5	4	9
5	M 3 m	5.6	Yes	Ic	2	175	0.2	5	5	17
6	M 1m 19d	5.7	Yes	Ia	5.5	220	1	10	4	7
7	M 3m	6	No	Ic	2.2	220	0.3	5	4	7
8	F 4m 24d	6	Yes	Ia	4	160	1	5	5	8
9	F 1m 23d	4.1	Yes	Ia	2	170	1	5	4	8
10	F 2m 2d	5	Yes	Ia	7	150	1	3	4	11

None of the patients experienced complications. m, Month; D, day.

The patient was placed in the supine position and, after endotracheal intubation under general anaesthesia, pneumoperitoneum was established through the umbilicus. An 8-mm trocar was inserted into the umbilical incision. Another two 8-mm trocars were placed in the left upper and right lower abdomen between 3 and 8 cm from the umbilical incision, and a 5-mm laparoscopic trocar was placed in the left abdomen as an auxiliary port (*Fig. 1a*,*b* ). The jejunal Roux limb was fashioned extracorporeally through the enlarged umbilical incision, then the umbilical wound was partially closed to refit the 8-mm trocar. Adjusting the patient’s position to head high and feet low, after setting the upper abdominal operation mode of the machine, the no. 3 robotic arm was connected to the umbilical trocar and robotic arms no. 2 and 4 were connected to the right lower and left upper abdominal trocars. Suspending the gallbladder fossa and ligamentum teres hepatis through the abdominal wall, the gallbladder was removed from the gallbladder fossa with the electric hook. If the cyst was large, decompression was performed first to increase the operating space, then the cysts were dissected using an electric hook, keeping close to the cyst wall and away from the portal vein and hepatic artery (*Fig. 1c*). The dissection continued down to the distal aspect of the choledochal cyst to the point where it tapered, then the distal end was ligated with Hem-o-Lok^®^ (Hangzhou, China) polymer clips (*Fig. 1d*). The proximal end of the cyst was dissected in reverse along the cyst wall to the hepatic duct of the hilar part (*Fig. 1e*). A 4/0 absorbable suture was used for end-to-side hepaticojejunostomy (*Fig. 1f*). After the surgery, a drainage tube was placed around the anastomotic site, then the choledochal cyst and gallbladder were removed through the umbilical incision. All 10 patients recovered well after the operation with no complications.

**Fig. 1:**
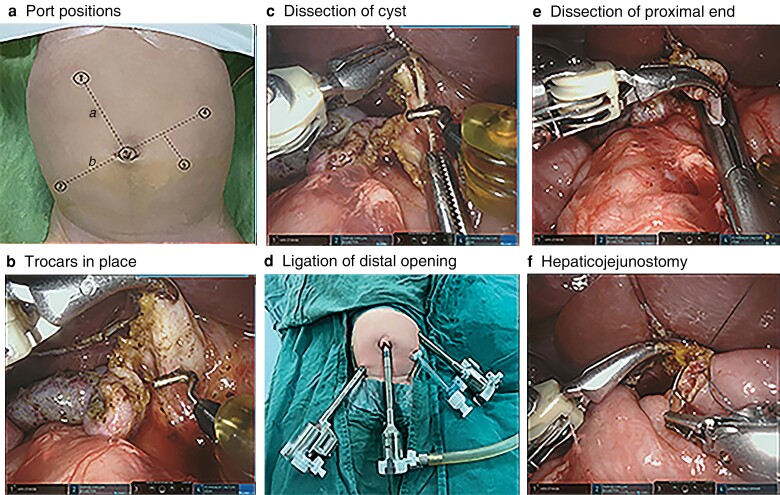
Robot-assisted resection of choledochal cysts **a**,**b** Positioning of ports. The umbilicus and right upper abdomen with a hepatic hilum body surface projection were taken as two points to make line *a*, then line *b* was drawn perpendicular to line *a* through the umbilicus (point 3). Two points (2 and 4) were marked on line *b* about 3–8 cm away from the umbilicus; points 2, 3, and 4 were the positions of the Da Vinci 8-mm trocar, and point 5 the position of the 5-mm accessory port for the assistant surgeon. **c** Dissection of the cyst keeping close to the cyst wall. **d** Ligation of the distal opening with a Hem-o-Lok polymer clip. **e** The proximal end of the cyst was dissected to the hepatic duct of the hilar part. **f** End-to-side hepaticojejunostomy.

## Data Availability

The authors are willing to make their data, analytical methods, and study materials available to other researchers, who can contact ebwk@zju.edu.cn to obtain these materials.
